# Embryonic thermal manipulation reduces hatch time, increases hatchability, thermotolerance, and liver metabolism in broiler embryos

**DOI:** 10.1016/j.psj.2024.103527

**Published:** 2024-02-01

**Authors:** Sadid Al Amaz, Md Ahosanul Haque Shahid, Ajay Chaudhary, Rajesh Jha, Birendra Mishra

**Affiliations:** Department of Human Nutrition, Food and Animal Sciences, College of Tropical Agriculture and Human Resources, University of Hawaii at Manoa, Honolulu, Hawaii, 96822

**Keywords:** broiler, embryo, metabolism, thermal manipulation, thermotolerance

## Abstract

The broilers’ health and growth performance are affected by egg quality, incubation conditions, and posthatch management. Broilers are more susceptible to heat stress because they have poor thermoregulatory capacity. So, it is crucial to develop a strategy to make chicks thermotolerant and cope with heat stress in post-hatch life. This study investigated the effects of embryonic thermal manipulation (**TM**) on different hatching parameters (hatch time, hatchability, and hatch weight), brain thermotolerance, and liver metabolism. Six hundred fertile Cobb 500 eggs were incubated for 21 d. After candling on embryonic day (**ED**) 10, 238 eggs were thermally manipulated at 38.5°C with 55% relative humidity (**RH**) from ED 12 to 18, then transferred to the hatcher (ED 19–21, standard temperature, 37.5°C) and 236 eggs were incubated at a standard temperature (37.5°C) till hatch. The samples were collected from the Control and TM groups on ED 15 and 18 of the embryonic periods. Hatchability was significantly higher (*P* < 0.05) in the TM group (94.50%) than in the control group (91.0%). Hatch weight did not differ significantly between the TM group (50.54 g) and the Control group (50.39 g). Most importantly, hatch time was significantly lower (*P* < 0.05) in the TM group than in the Control. In the D15 embryo brain, the mRNA expression of ***TRPV1****,****TRPV2, TRPV3****,* and the epigenetic marker ***H3K27*** were significantly lower (*P* < 0.05) in the TM group compared to the Control group. However, in the D18 brain, the expression of ***TRPV1, TRPV2***, and ***CRHR1*** was significantly higher (*P* < 0.05) in the TM group than in the Control group. In the liver, the mRNA expression of ***SLC6A14*** was significantly lower (*P* < 0.05) in the D15 TM group than in the D15 Control group. Conversely, the ***DIO3*** mRNA expression was significantly higher (*P* < 0.05) in the D15 TM group than in the D15 Control group. The expression of ***GPX3, FOXO1, IGF2***, and ***GHR*** in the liver was significantly higher in the D18 TM group compared to the D18 Control group (*P* < 0.05). In conclusion, increased expression of the aforementioned markers during the later embryonic period has been linked to reduced hatch time by increasing liver metabolism and thermotolerance capacity in the brain.

## INTRODUCTION

The demand for poultry meat is expected to increase in the coming decades due to the global rise in the human population (Michele, 2023). To achieve sustainable high-production efficiency in the supply chain, hatcheries are required to optimize the hatchability of healthy chicks, ensuring high survival rates and maximal growth potential. Hatching parameters (hatch time, hatchability, and hatch weight) are essential for the poultry industry. Artificial incubation relies on the precise control of environmental factors, such as temperature, relative humidity, turning, and oxygen availability, to assure satisfactory embryo development and allow maximum productivity (Tona et al., 2022). Therefore, environmental temperature affects hatchability, embryonic development, chick quality, and posthatch bird performance. An effective incubation strategy is necessary to promote embryo development and reprogramming, positively impacting the broiler's hatchability, thermotolerance, and metabolism (Loyau et al., 2014).

The embryonic thermal manipulation (**TM**) strategy has been tested with promising results on hatch time, hatchability (Piestun et al., 2008a), and hatch weight (Yahav et al., 2004). TM has been shown to mitigate the adverse effects of posthatch heat stress by improving thermotolerance acquisition during embryogenesis (Al Amaz et al., 2024). Higher thermotolerance can be achieved by rapid thermal stress response, acclimation, and epigenetic temperature adaptation (Yahav, 2009). Epigenetic temperature adaptation regulates gene expression that does not hinge on gene sequence, can occur during early pre or postnatal development, and may result in long-term physiological memory (Nichelmann and Tzschentke, 2002). In broilers, TM during embryogenesis improved thermotolerance procurement by imposing epigenetic temperature adaptation in the embryonic stage (David et al., 2019). However, it is most effective during the critical period of hypothalamus-hypophysis-thyroid or adrenal axis development or both (Piestun et al., 2008b). The early TM affects avian embryos' hatch parameters and posthatch development by regulating their metabolism. Around 80% into the incubation period, the metabolic rate of the embryo reaches a plateau in precocial bird species. This plateau lasts approximately 2 d (17–18) in the poultry (Dietz et al., 1998). There is some evidence of increased posthatch metabolism due to embryonic thermal conditioning. TM at 39°C and 65% RH for 12 h/d during embryonic days (**ED**) 7 to 16 significantly influenced thyroid hormone metabolism by lowering the muscle mRNA regulation of Iodothyronine Deiodinase (**DIO**). TM increased energy utilization in the liver (Loyau et al., 2014). However, there are limited studies on the effects of TM on embryo metabolism in the critical stages of embryogenesis. Yet, the relationship between hatchability and liver metabolic activity remains elusive in broiler chickens.

Therefore, based on the functionality of TM, we hypothesized that TM would increase the broiler's thermotolerance capacity and liver metabolism, which would positively impact the hatching performances. To delineate the detailed mechanism by which TM modulates the physiology of embryos, we analyzed several markers related to growth and development, stress, and antioxidants in the brain and liver tissue. These markers serve as essential indicators for potential factors that can influence the development of tissues/organs. Based on the published reports and our extensive experiences with thermal stress in chickens, we selected a series of markers related to heat shock protein-related genes (*HSF3, HSP70, HSPH1*, and *HSPD1*, antioxidants (*SOD1, SOD2, TXN, GPX1, GPX3,* and *NFE2L2)* (Al-Zghoul, 2018), nutrient transporters (*SLC3A1* and *SLC6A14*) and metabolism (*FBP1, ACP6, FOXO1,* and *DIO3)* (Wang et al., 2014), and growth-related genes (*IGF1, IGF1R, IGF2,* and *GHR*) (Loyau et al., 2014) in the liver. To comprehensively understand the TM in the brain, we analyzed some key markers for heat shock protein-related genes, antioxidants, thermoregulation (*TRPV1, TRPV2, TRPV3, TRPA1, Eif2b45, CREB1, CRHR1,* and *CRHR2)*, and epigenetics (*DNMT3A, DNMT3B, TDG, Gadd45B, EZH2, and H3k27)* (Xu et al., 2022).

## MATERIALS AND METHODS

### Experimental Design

**Figure 1:**
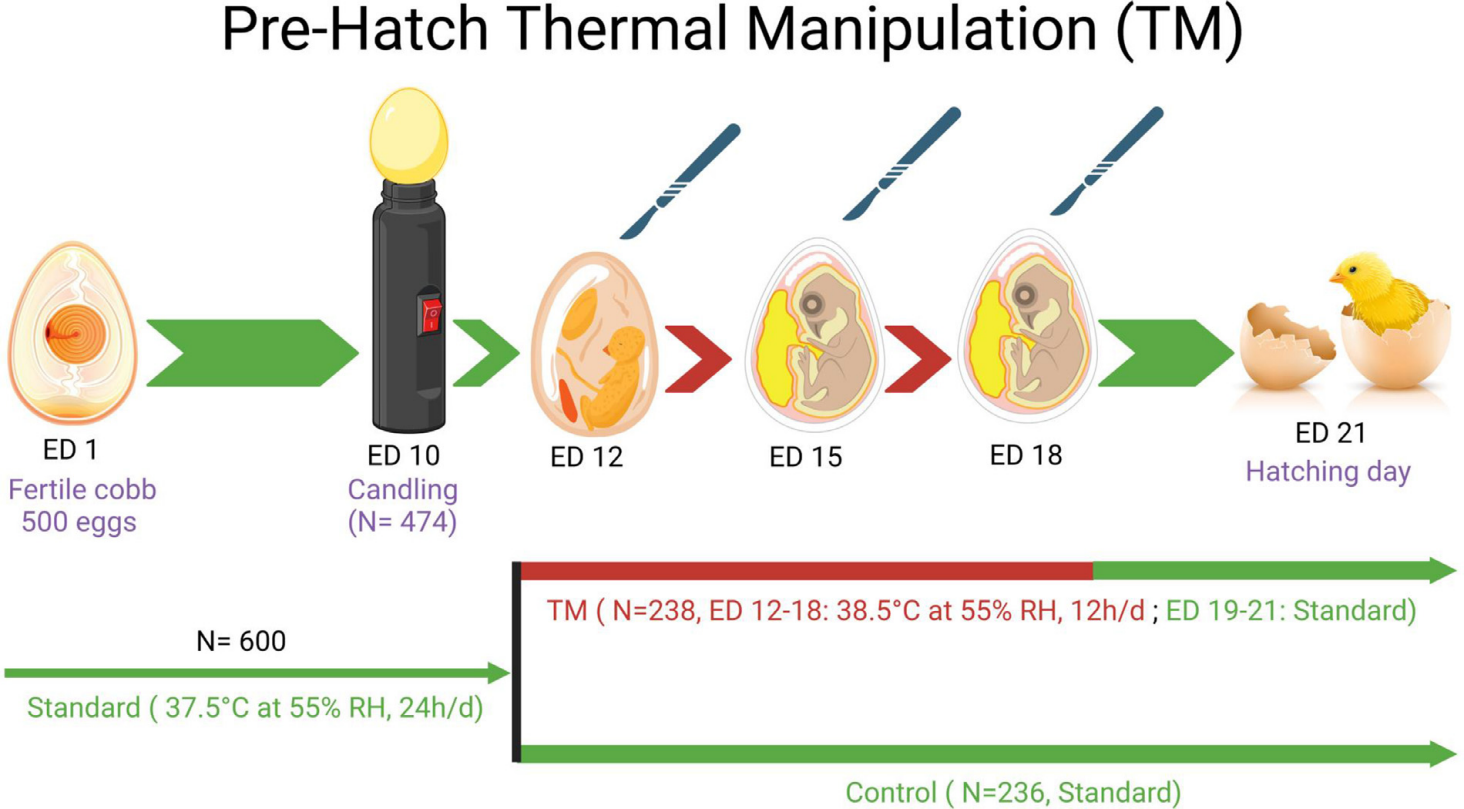
Schematic diagram of prehatch thermal manipulation (TM).

The University of Hawaii Institutional Animal Care and Use Committee (**IACUC**) approved all animal experimentation procedures (Approval No 17-2605-6). Fertile Cobb 500 eggs (n = 600) were sourced from a local Hatchery (Asagi Hatchery Inc, Honolulu, HI) for embryonic TM. All the eggs were incubated in 3 incubators (GQF incubator, Savannah, GA; 200 eggs each) at standard temperature (37.5°C at 55% RH, 24h/d) for the first ED 11. The candling was done on ED 10 to separate the live embryos (n = 474). On ED 12, eggs were divided into 2 groups: 1) Control (n = 236) (37.5°C at 55% RH, 24 h/d until the hatch day, ED 21), and 2) TM group (n = 238; 38.5°C at 55% RH, 12 h/d, from ED 12 to ED 18 and standard temperature from ED 19–ED 21) in 2 incubators for each group with automatic temperature control, 55% RH, and egg turning (every 2 h), as shown as Figure 1. Studies have explored the impact of TM between 38-40°C on embryonic health and production (Al-Zghoul and El-Bahr, 2019). According to the findings, maintaining a temperature within the range of 38°C to 39°C positively affected health and production. However, the temperature exceeding 39.5°C or 40°C negatively affected hatchability and increased embryo mortality. Based on these findings, we opted for a TM of 38.5°C during the critical phase of embryonic development (ED 12–18) to investigate the hatch parameters and the underlying mechanisms.

### Sample Collection

Embryonic brain and liver tissue were collected from the Control and TM groups on ED 15 and ED 18 (n = 6) prior to commencing the TM on each sampling day. First, the egg was broken, and then the embryos were euthanized using carbon dioxide asphyxiation for sampling. The whole brain and liver were collected, snap-frozen, and stored at −80°C until RNA extraction.

### Hatching Parameters

Hatch time, hatchability, and hatch weight were calculated right after hatching. Hatchability was measured by percentage (total number of chicks hatched/total fertile eggs). There were (n = 4) egg trays for both the Control and TM groups that were used as replicates for determining the hatchability (%) and hatch time (h). All chicks were weighed individually from the TM and Control group. Hatch time was measured by hours. We measure hatch time by hours. We started incubation at 11 am on the first day and counted the next day at 11 am as 24 h. With the goals to calculate the hatch time, we meticulously tracked the hatching until the first bird from each treatment group emerged from the eggshell. We stopped counting when the first bird hatched in each treatment group. There were 4 trays of eggs in the incubator. Around 75% of the birds hatched from 3 of the trays before the first bird of the control group hatched. However, there was an overlap of 1 h in the last tray with the control group. Further, we calculated the hatching time by averaging each tray.

### Quantitative Real-Time PCR (**qPCR**)

TRIzol reagent (Invitrogen, Carlsbad, CA) was used to isolate total RNAs from frozen tissues (50–100 mg) according to the manufacturer's instructions. The concentration of total RNA was measured using NanoDrop One (Thermo Fisher Scientific, Madison, WI). The quality of the RNA was determined by running samples through 2% agarose. The RNA samples were kept at -80°C until further analysis. The expressions of candidate genes were analyzed using qPCR (Quant Studio 3, Applied Biosystems, Foster City, CA) as described previously (Chaudhary et al., 2023). Specific primer pairs for detecting each gene were designed using the NCBI Primer-Blast tool. Using a High-Capacity cDNA Reverse Transcription Kit (Applied Biosystems, Foster City, CA), 1 μg of total RNA was reverse-transcribed into complementary DNA (**cDNA**) and then diluted with nuclease-free water (1:25). qPCR was performed using a Real-time PCR and PowerUp SYBR Green Master Mix (Applied Biosystems, Foster City, CA). The final volume of the qPCR reaction mixture was 10 μL, comprised of 3 μL of cDNA, 5 μL of PowerUp SYBR Green Master Mix, and 1 μL of each forward and reversed primer at a concentration of 5 μmol. Standard cycling mode was used for the qPCR reaction. For validating the SYBR Green-based objective amplicon, a melting curve was constructed. In addition, the specificity of each primer pair was determined through 1% gel electrophoresis of the qPCR products. In triplicate, 3 housekeeping genes were analyzed*: glyceraldehyde 3-phosphate dehydrogenase* (**GAPDH**), *beta-actin* (**ß-actin**), and *TATA-box binding protein* (**TBP**). The *TBP* expression was consistently stable across the brain tissues, while *GAPDH* yielded the most stable results for the liver. The target genes were analyzed twice, and the average value of each experimental replicate was determined. The expression levels of target genes were quantified using cycle threshold (**Ct**) values normalized with *TBP* for the brain and *GAPDH* for the liver. Using the 2^¯ΔΔCt^ method, the fold change of every gene was calculated. The primers are presented in Supplementary Table 1.

### Statistical Analyses

The gene expression data were analyzed using GraphPad (GraphPad Software, San Diego, CA). An unpaired T-test was performed to analyze the hatch time, weight, and hatchability, as there were only 2 treatment groups until the hatch. Gene expressions were analyzed using a 2-way analysis of variance (**ANOVA**), followed by the Tukey-HSD test for mean separation. All data are presented as mean ± SEM. The statistical significance threshold was set at *P <* 0.05.

## RESULT

### Hatchability Variables

**Figure 2:**
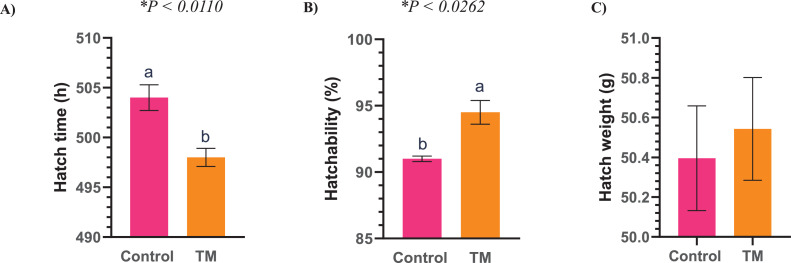
Effects of TM on hatching parameters. (A) Hatch time (h), (B) Hatchability (%), and (C) Hatch weight (g). Different letters indicate a significant difference (*P* < 0.05) between the treatment groups.

The hatch time (h), hatchability (%), and hatch weight (g) are presented in Figure 2. The hatchability rate was significantly higher (*P* < 0.05) in the TM treatment group (94.50%) compared to the Control group (91.0%). Hatch weight did not differ significantly between the TM group (50.54 g) and the Control group (50.39 g). Moreover, hatch time was significantly lower (*P* < 0.05) in the TM group (498 h) compared to the Control group (504 h).

### Effects of TM on the Brain Gene Expression

**Figure 3:**
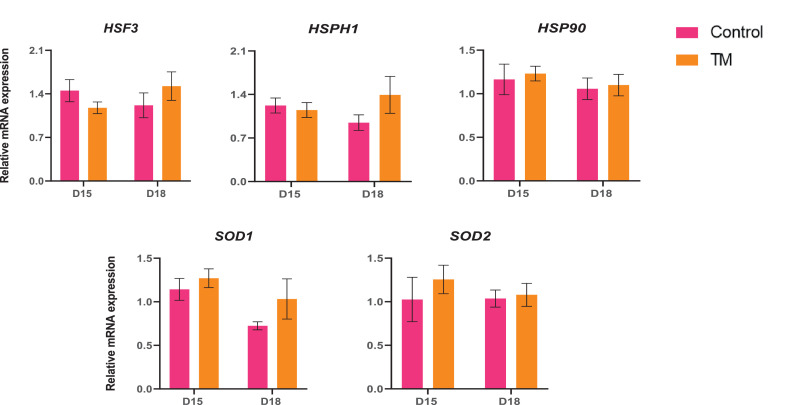
Effects of TM on the mRNA expression of heat shock and antioxidant genes in the brain. Data presented as mean ± SEM. Different letters indicate a significant difference (*P* < 0.05) among the treatment groups.

The expression pattern of the heat shock protein-related genes (*HSF3, HSPH1,* and *HSP90*) and the antioxidant genes (*SOD1* and *SOD2*) among the treatment is shown in Figure 3. The expressions of *HSF3, HSPH1*, and *HSP90* mRNA were relatively higher in the D18 TM group compared to the D18 Control (*P* > 0.05). The expression of *SOD1* and *SOD2* were improved in the D15 TM and D18 TM groups than in the D15 Control and D18 Control groups (*P* > 0.05).

**Figure 4:**
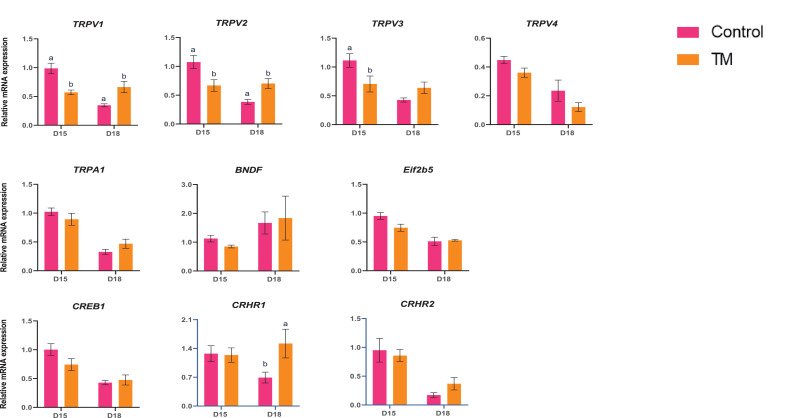
Effects of TM on the brain's mRNA expression of thermoregulation genes. Data presented as mean ± SEM. Different letters indicate a significant difference (*P* < 0.05) among the treatment groups.

The expression of the thermoregulatory genes (*TRPV1, TRPV2, TRPV3, TRPA1, Eif2b45, CREB1, CRHR1,* and *CRHR2*) is presented in Figure 4. *TRPV1, TRPV2, and TRPV3* mRNA expression were significantly lower (*P* < 0.05) in D15 TM compared to D15 Control. However, *TRPV1, TRPV2,* and *CHRH1* gene expression were significantly higher (*P* < 0.05) in the D18 TM group compared to the D18 Control group.

**Figure 5:**
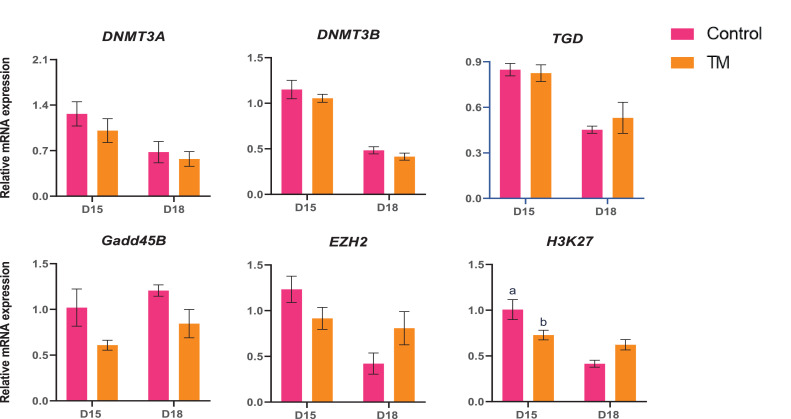
Effects of TM on the mRNA expression of epigenetic markers genes on the brain. Data presented as mean ± SEM. Different letters indicate a significant difference (*P* < 0.05) among the treatment groups.

The mRNA expression of the epigenetics markers (*DNMT3A, DNMT3B, TDG, Gadd45B, EZH2, and H3k27*) is presented in Figure 5. *H3K27* mRNA expression was significantly lower (*P* < 0.05) in the D15 TM than in the D15 Control group.

### Effects of TM on Liver Gene Expression

**Figure 6:**
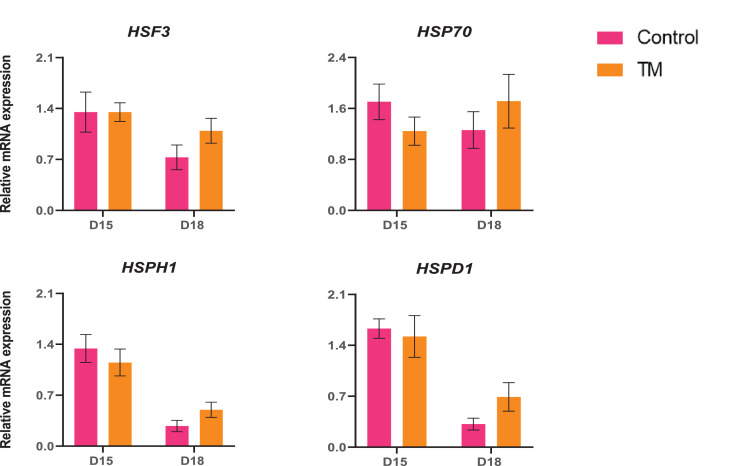
Effects of TM on the mRNA expression of heat shock genes on the liver. Data presented as mean ± SEM. Different letters indicate a significant difference (*P* < 0.05) among the treatment groups.

The expression patterns of heat shock proteins-related genes (*HSF3, HSP70, HSPH1*, and *HSPD1*) were significantly unchanged (*P* > 0.05) as shown in Figure 6.

**Figure 7:**
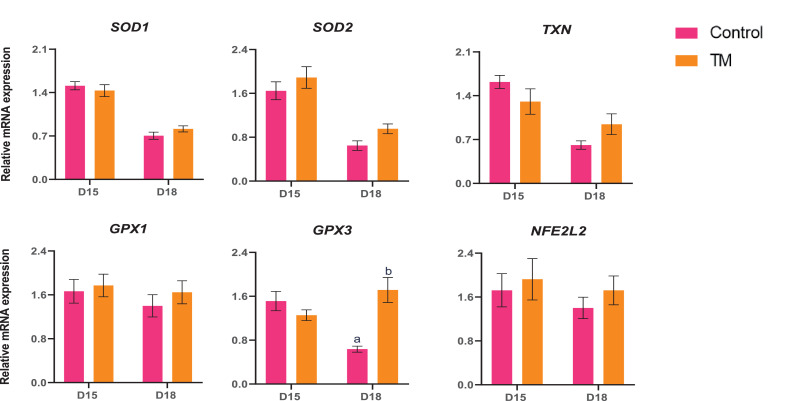
Effects of TM on the liver's mRNA expression of antioxidant genes. Data presented as mean ± SEM. Different letters indicate a significant difference (*P* < 0.05) among the treatment groups.

The expression of antioxidant genes (*SOD1, SOD2, TXN, GPX1, GPX3,* and *NFE2L2*) expression are presented in Figure 7. The expression of *GPX3* was significantly higher (*P* < 0.05) in the D18 TM than in the D18 Control group. However, the expression of *SOD1, SOD2, TXN, GPX1,* and *NFE2L2* did not show any significant changes.

**Figure 8:**
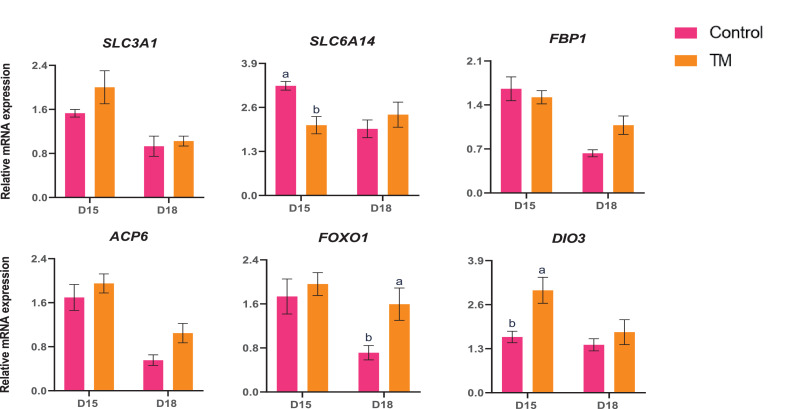
Effects of TM on the mRNA expression of nutrient transporter and metabolism-related genes in the liver. Data presented as mean ± SEM. Different letters indicate a significant difference (*P* < 0.05) among the treatment groups.

The mRNA expression of nutrient transporter and metabolism-related genes (*SLC3A1, SLC6A14, FBP1, ACP6, FOXO1,* and *DIO3*) are presented in Figure 8. The expression of *SLC6A14* was significantly lower (*P* < 0.05) in D15 TM than in the D15 Control group. The expression of *FOXO1* was significantly higher (*P* < 0.05) in D18 TM than in the D18 Control. *DIO3* expression was significantly higher (*P* < 0.05) in D15 TM than in D15 Control. However, TM improved the expression of *SLC3A1* and *ACP6* in both embryonic D15 and D18 (*P* > 0.05).

**Figure 9:**
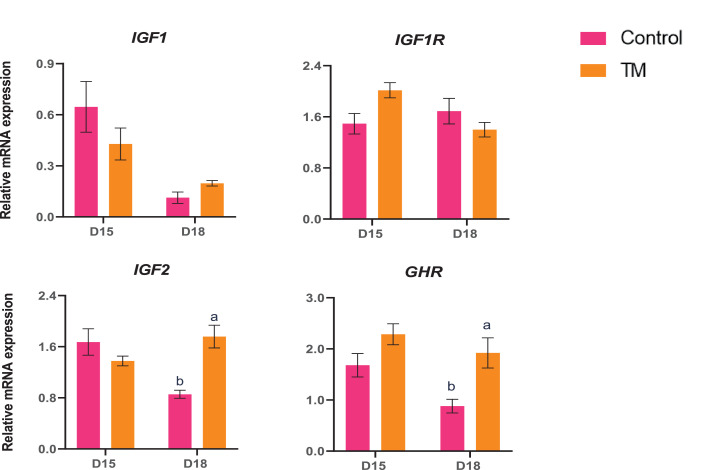
Effects of TM on the mRNA expression of growth and growth hormone-related genes in the liver. Data presented as mean ± SEM. Different letters indicate a significant difference (*P* < 0.05) among the treatment groups.

The expression of growth and growth hormone-related genes (*IGF1, IGFR, IGF2,* and *GHR*) are presented in Figure 9. The expressions of *IGF2* and *GHR* was significantly higher (*P* < 0.05) in D18 TM than in D18 Control. However, IGF*1* and *IGF1R* mRNA expression remained (*P* > 0.05).

## DISCUSSION

In poikilotherm embryos, deviations from the optimal incubation temperature range from 37°C to 37.50°C (Decuypere and Michels, 1992) may impact embryo size, organ and skeletal growth, metabolism, physiological growth and development, and hatching rate (Black and Burggren, 2004). The temperature, embryonic stage, and timing should be synchronized to achieve the perfect TM. Short-term incubation temperature simulation (38.20°C–38.40°C, 2 h daily from ED 18–ED 21) has a higher hatchability (Tzschentke and Halle, 2009). However, TM at 39.5°C and 65% RH for 12 h from ED 7 to ED 16 significantly increased embryo mortality and decreased hatchability compared to the embryos at 37.5°C and 56% RH (Brannan et al., 2021). So, precise temperature simulations are critical. In this study, TM significantly increased the hatchability by 3.50% than in the control group. Interestingly, hatch time was significantly decreased by 6 h in the TM group than in the control group. Therefore, TM at 38.5°C from ED 12 to 18 for 12 h/d at 55% RH positively impacted the important hatching parameters. Even a marginal improvement in hatchability can yield significant economic benefits for the commercial broiler industry. By reducing the duration of incubation, production costs and time can be significantly reduced.

Changes in incubation temperature induced epigenetic temperature adaptation in poultry (David et al., 2019). As a result of perinatal epigenetic temperature adaptation, neuronal thermosensitivity in the hypothalamus and peripheral thermoregulatory mechanisms are altered (Lindquist and Craig, 1988; Tzschentke, 2007). To understand the potential thermoregulatory mechanism of TM in embryonic stages, we analyzed some heat shock protein-related genes in the embryo's brain. Heat stress induces the synthesis of a group of heat-shock proteins (**HSPs**) in all organisms and plays a crucial role in repairing and preserving the internal environment by promoting the degradation of misfolded proteins and aiding in protein refolding. HSPs are an emergency response, and their induction is remarkably rapid and intensive. The strong correlation between the induction of HSPs and the development of thermotolerance demonstrates that HSPs have a protective function (Lindquist and Craig, 1988). *HSF3* is the master regulator of HSP expression in the avian species (Sarge et al., 1993). Higher expression of *HSF3* might be responsible for expressing *HSPH1 (HSP110)* and *HSP90* in the D18 TM group compared to the D18 Control group. *HSPH1* is regarded as an antiapoptotic and proautophagy factor that regulates apoptosis, development, cell motility, autophagy, and the adaptability of cells to stress (Stürner and Behl, 2017). So, our results suggest that TM induced upregulation of *HSF3* in D18 TM embryos, which helps to increase the gene expression of *HSPH1* and *HSP90.* Increasing gene expression of *HSPH1* and *HSP90* might help in thermoregulation and reduce apoptosis during embryogenesis, supporting the lower embryo mortality rate in the TM embryos. Chick embryonic tissues require an antioxidant defense (Surai, 2016) due to the large concentration of highly polyunsaturated fatty acids in the lipid fraction (Speake et al., 1998). *SOD1* and *SOD2* are considered the first line of antioxidant defense mechanisms. In chicken embryonic tissues, they offer efficient defense against lipid peroxidation. SOD production under stress is an adaptive mechanism to reduce reactive oxygen species (**ROS**) generation. When the stress level is too high, the SOD activity is reduced, triggering apoptosis (Surai, 2016). This study revealed increasing *SOD1* and *SOD2* expression in both D15 TM and D18 TM groups. Increased SOD expression might help the embryo to respond better at the higher incubation temperature without hampering embryonic growth.

It is reported (Dhahir, 2016) that chicks exposed to heat acclimation during the late hatching and early postnatal period could develop superior heat tolerance in later life. The activation of the hypothalamic-pituitary-adrenal (**HPA**) axis in the chicken embryo is thought to occur during the mid or late-embryonic period. At this stage, raising the incubation temperature could cause associated genes to undergo epigenetic changes that alter the threshold for the hypothalamus temperature set-point, changing chickens' response to heat stress. Transient receptor potential vanilloids (**TRPV**) receptors are extremely sensitive to heat stimulation (Ohya et al., 2016). TRP ion channels were mostly studied in mice, but very few in chickens known to be associated with heat acclimation mitigate the severe effects of heat stress via TRP ion channels, and epigenetics may facilitate this process. Ca^2+^ is a crucial indicator of heat acclimation, and TRPV genes may regulate Ca^2+^ transport in various organs via DNA methylation, thereby fostering the formation of heat acclimation, (Wu et al., 2020). Our results showed a significant upregulation (*P* < 0.05) of *TRPV1* and *TRPV2* in the D18 TM group compared to the D18 Control group. Higher expression of these genes might indicate that the embryos may develop heat acclimation by D18, thus increasing thermotolerance capacity On the other hand, *TRPV4* expression decreased in D18 TM embryos because its expression mostly depends on the inflammatory pathways (Aghazadeh et al., 2021). The production of H_2_O_2_ during the thermal stress induces reversible covalent modifications in cysteine residues of particular proteins, such as TRPV channels, and alters the activation state of the proteins (Belhadj-Slimen et al., 2014). In the hypothalamus, the neuronal network for *BDNF* (brain-derived neurotrophic factor) regulates eating, adaptive thermogenesis, and physical activity. *BDNF* neurons control feeding and adaptive thermogenesis in the anterior and posterior regions of the PVN, respectively (An et al., 2015). In mice, *BNDF* is beneficial for identifying warm-activated thermoregulatory neurons (Tan et al., 2016). The establishment of temperature control and the formation of taste preferences depend on the translation machinery's *Eif2b5* transcription regulation (Tirosh et al., 2007). In this study, the upregulation of both *BNDF* and *Eif2b5* on D18 TM indicates that the TM embryos were more thermotolerant than the D18 C embryos. In the brains of animal species, a transcription factor called cyclic AMP-responsive element binding protein (**CREB**) controls a wide range of cellular processes, including neuronal differentiation, cell survival, and memory formation (Lonze and Ginty, 2002). For chicks to develop heat tolerance throughout crucial stages of postnatal development, alterations in the histone methylation of the *BDNF* promoter in the hypothalamus are required. This may be because *BDNF*, which stimulates CRH expression through the TrkB (tropomyosin-related kinase B) or CREB (cAMP response-element binding protein) signaling pathway and subsequently has long-term effects on the HPA axis, is a potential target of CRH neurons in the hypothalamus of chickens (Kisliouk and Meiri, 2009). So, higher expression of *CREB1* may help express *BNDF* and a long-term memory formation in the embryos that might help improve thermotolerance in broilers' posthatch life. Corticotropin-releasing hormone (**CRH**), generated in the paraventricular nucleus (**PVN**), has a major negative feedback effect on the HPA axis. Heat stress enhances the HPA axis's ability to produce CRH, which increases the amount of glucocorticoids (corticosterone) in the body. Excess glucocorticoids also negatively regulate the HPA axis's reactivity. The *CRHR1* and *CRHR2* receptors simulate the release of adrenocorticotrophic hormone (**ACTH**), which stimulates the adrenal cortex to release glucocorticoid (**GC**) hormones, cortisol in humans, and corticosterone in rodents (Jawahar et al., 2015). So, based on those studies, significantly higher (*P* < 0.05) expression of *CRHR1* and increased *CRHR2* expression in our study means they help regulate the ACTH secretion more efficiently, thus enabling thermal acquisition more quickly in the heat stress condition. However, the downregulation of all the thermoregulatory genes in the D15 TM might be due to the embryo's thermal sensitivity. Embryos might not acclimatize to the elevated temperature within that short time. Thus, gene expression was impaired in the TM embryos. More studies are needed to comprehend the thermotolerance mechanism of the prehatch TM embryos.

Changes in cellular characteristics in the frontal hypothalamus occur during a crucial phase that leads to the development of thermal control establishment. The epigenetic coding that governs the repertoire of transcribed proteins may regulate these changes (Yossifoff et al., 2008). DNA methylation is a durable epigenetic marker that can be passed from generation to generation through various cell divisions without affecting the actual genetic codes. DNA methylation is dynamic during cell development and differentiation (Kim and Costello, 2017). Typically, the promoter CpG dinucleotide location is where most DNA methylation occurs. The 5-carbon position of cytosine covalently links to a methyl group to create 5-methylcytosine (**5mc**) in the presence of DNA methyltransferase (**DNMT**) (Cramer et al., 2019). *DNMT3a* and *DNMT3b* are involved in de novo methylation (Goll and Bestor, 2005). It has been established that their transcription regulatory mechanism contributes to neural plasticity and long-term memory (Levenson et al., 2006). More importantly, the control of *BNDF* expression during the development of thermotolerance involves intricate and dynamic alterations in DNA methylation (Yossifoff et al., 2008). In this study, the downregulation of *DNMT3A* and *DNMT3B* in D18 TM than in D18 Control might indicate a higher thermal acquisition by regulating higher *BNDF* gene expression. *TDG* regulates de novo DNMT activities to prevent improper methylation, and its association with XRCC1 and APE1 suggests it functions via a base excision repair (Cortázar et al., 2011). The upregulation of *TDG* might aid the DNMT expression in the embryos in the D18 TM group compared to the D18 Control group. During the growth, the growth arrests DNA damage (*Gadd45*) is involved in DNA demethylation and in controlling DNA damage repair, cell proliferation, cell apoptosis, and the cell cycle. Specifically, Gadd45b supports *BDNF* promoter demethylation by allowing the removal of 5HMC (Gavin et al., 2015). So, the downregulation of *Gadd45b* on D15 TM and D18 TM compared to their control groups in this study may help DNA methylation, thus paving the way to express *BNDF* more efficiently. The expression of *H3K27* and *EZH2* genes are interconnected. The enzyme Enhancer of Zeste 2 (***EZH2***) is a component of Polycomb Repressive Complex 2 (***PRC2***), which catalyzes the lysine 27 trimethylation (*H3K27me3*) of histone H3 at target promoters to silence genes. Gene activation or repression is caused by substantial *EZH2* occupancy at promoters indicated by either H3K27ac or H3K27me3, respectively (Kim et al., 2018). *EZH2* is essential for establishing heat acclimation and responding to heat stress, resulting in heat susceptibility (Geranton, 2019). Thus, the higher expression of *H3K27 in* the D18 TM group than the D18 Control group in this study may help to express higher mRNA expression of *EZH2*, which might help heat acclimation of the broilers in later life. However, the upregulation phenomenon of *DNMT3B* and downregulation of *TDG, H3K27,* and *EZH2* can be explained by the short heat acclimatization period of the embryo in D15, as the TM started in D12. So, the embryos might take time to familiarize themselves with the elevated incubation temperature.

Unlike the brain, we also analyzed some HSPs in the liver. *HSP70* is one of the most studied heat shock proteins. *HSP70* acts as a chaperone during cellular stress events and triggers the expression of several inflammatory cytokines recognized as critical factors during early liver regeneration (Wolf et al., 2014). *HSPD1* performs crucial roles in protecting cells and tissues against heat stress. It can induce cellular inflammation in response to stressful conditions, thereby protecting cells (Siddiqui et al., 2022). Thus, higher expression of *HSP70* and *HSPH1* in our study suggests that they play a crucial role in protecting against cellular damage in the metabolically active embryonic liver, thus achieving a lower mortality rate of the embryos.

Embryo development necessitates more oxygen to provide energy. However, elevated oxygen levels result in elevated ROS levels (Turrens, 2003). ROS induces myocardial hypertrophy in developing chick embryos (Li et al., 2014). Throughout the incubation period, SOD metabolizes extremely reactive superoxide anions into H_2_O_2_ and O_2_. GPX catalyzes the reduction of hydrogen and lipid hydroperoxides using glutathione as the electron donor. It degrades H_2_O_2_ and other peroxides (Yang et al., 2018). *TXN* restores the enzymatic activity of ROS-damaged proteins, such as oxidized Prdx, by reducing oxidized active site cysteines (Fernando et al., 1992). *NFE2L2* (*Nrf2*) is a regulator of cellular oxidant resistance. To regulate the physiological and pathophysiological effects of oxidant exposure, it triggers the expression of an array of antioxidant response element–dependent genes (Ma, 2013). In our study, significantly higher (*P* < 0.05) *GPX3* mRNA expression and numerically higher other antioxidant gene expression in D18 TM than D18 Control supports previous studies. Higher antioxidant gene expression might play a vital role in achieving a higher hatch body weight and hatching rate in TM embryos.

During embryonic development, the chick is nourished by the yolk via the yolk-sac membrane and derives nutrients for the intestinal cells from the bloodstream via the basolateral surface (Speake et al., 1998). *SLC3A1* encodes a neutral and basic protein called rBAT that acts as an amino acid transporter (Lee et al., 1993). *SLC6A14* transports 18 proteinogenic amino acids, including all neutral and cationic amino acids (Nakanishi et al., 2001). Fructose-1, 6-bisphosphatase 1 (***FBP1***), is a crucial enzyme in gluconeogenesis, capable of transferring *FBP1* into fructose-6-phosphate(Lovtrup-Rein, 1989). ACP6 is a mitochondrial phosphate phosphatase that regulates lipid metabolism by hydrolyzing lysophosphatidic acid into a monoacylglycerol (Gao et al., 2022). *FOXO1* is a primary transcriptional regulator of gluconeogenesis, is inversely regulated by insulin, and exerts considerable effects on hepatic lipid metabolism (Tikhanovich et al., 2013). Thyroid hormone (**TH**) deiodinases are crucial in the functional diversification of TH signaling. *DIO3* is involved in the development, growth, and metabolic processes and regulates TH homeostasis in a cell-specific manner (Sabatino et al., 2021). *DIO3* deficiency in mice results in various neurodevelopmental and behavioral abnormalities, demonstrating the deleterious effects of TH excess and highlighting the essential function of *DIO3* in regulating TH action in the brain (Hernandez and Stohn, 2018). Our results showed an increased mRNA expression of *SLC3A1, AP6, FOXO1,* and *DIO3* in the D15 TM and D18 TM embryo's livers compared to their control group. The expression of *SLC6A14* and *FBP1* was also increased in the D18 TM liver than in the D18 C group. Taken together, the higher expression of the nutrient transporter and metabolic genes showed an increased metabolism in the TM embryos, which might be the reason for the early hatch time in thermally manipulated embryos.

Embryonic growth is crucial during embryogenesis because poor embryonic growth can reduce the neonatal chick, impacting growth and development during the posthatch period. *IGF1* and *IGF2* play significant roles in cellular growth by regulating growth hormone mechanisms. They can influence various biological processes in poultry, including growth, differentiation, and reproduction (McMurtry et al., 1997). Extensive research has demonstrated that *IGF1* and *IGF2* are associated with body weight and carcass traits (Wang, 2005; Zhou et al., 2005). Our study showed a significant decrease in *IGF1* expression in D15 TM than in the D15 C embryos. However, *IGF1* expression was higher in D18 TM than in the D18 group, suggesting heat acclimatization and a higher growth rate in the thermally manipulated embryos. The growth hormone receptor (***GHR***) is associated with various phenotypic and physiological alterations in chickens, including decreased body mass and reduced muscle mass. *GHR* gene expression is essential for chicken mitochondrial function (Hu et al., 2019). The result showed a significant increase in the *IGF2* and *GHR* expression in D18 TM than in their D18 Control group. Thus, the increased expression of the aforementioned markers during the later embryonic period has been linked to reduced hatch time by increasing liver metabolism and thermotolerance capacity in the brain.

In conclusion, the embryonic TM positively impacted the markers for liver metabolism, resulting in higher growth and metabolism, shorter hatch time, and higher hatchability. These results indicate that the embryonic TM exhibits a greater degree of developmental efficiency compared to the control embryos. Additionally, TM enhanced the key markers for the thermotolerance capacity of the embryo. Further study is needed to fully comprehend the detailed mechanism in thermally manipulated embryos and posthatch growth potential.

## Appendix Supplementary materials

Image, application 1

## Footnotes

Supplementary material associated with this article can be found in the online version at doi:10.1016/j.psj.2024.103527.
