# Supplementary material for: Embryonic thermal manipulation reduces hatch time, increases hatchability, thermotolerance, and liver metabolism in broiler embryos
Source: Poult Sci. 2024 Feb 1;103(4):103527. doi: 10.1016/j.psj.2024.103527 (PMC10907853; doi:10.1016/j.psj.2024.103527)
Supplement: Supplementary file 1 [file mmc1.docx]

**Supplement Table 1:** List of primers used in qPCR.

| **Gene** | **Accession No.** | **Primer sequence** | **Amplicon length** |
| --- | --- | --- | --- |
| *TBP* | XM_025148547.3 | F: TAGCCCGATGATGCCGTAT | 147 |
|  |  | R: GTTCCCTGTGTCGCTTGC |  |
| *GAPDH* | NM_204305.2 | F: AGCTTACTGGAATGGCTTTCCG | 122 |
|  |  | R: ATCAGCAGCAGCCTTCACTACC |  |
| *B-Actin* | NM_205518.2 | F:GAG AAA TTG TGC GTG ACA TCA | 152 |
|  |  | R:CCT GAA CCT CTC ATT GCC A |  |
| *HSF3* | NM_001305041.1 | F: TTCAGCGATGTGTTTAACCCT | 244 |
|  |  | R: GGAGGTCTTTTGGATCCTCT |  |
| *HSPH1* | NM_001159698.2 | F: AAACTGATGGTCAGCAAACG | 239 |
|  |  | R: TTGCATGATCATCTTACCCTCT |  |
| *HSPD1* | NM_001012916.3 | F: AACAAAGGTTGTGAGAACGG | 161 |
|  |  | R: ATTAGAACATGCCACCTCCC |  |
| *HSP70* | NM­_001006685.1 | F: TCTCATCAAGCGTAACACCAC | 104 |
|  |  | R: TCTCACCTTCATACACCTGGAC |  |
| *HSP90* | NM_001109785.1 | F: GATAACGGTGAACCTTTGGG | 120 |
|  |  | R: GGGTAGCCAATGAACTGAGA |  |
| *SOD1* | NM_205064.1 | F: CAACACAAATGGGTGTACCA | 119 |
|  |  | R: CTCCCTTTGCAGTCACATTG |  |
| *SOD2* | NM_204211.1 | F: CCTTCGCAAACTTCAAGGAG | 160 |
|  |  | R: AGCAATGGAATGAGACCTGT |  |
| *TXN* | NM_205453.1 | F: GGCAATCTGGCTGATTTTGA | 79 |
|  |  | R: ACCATGTGGCAGAGAAATCA |  |
| *GPX1* | NM_001277853.2 | F: AATTCGGGCACCAGGAGAA | 101 |
|  |  | R: CTCGAACATGGTGAAGTTGG |  |
| *GPX3* | NM_001163232.2 | F: GAGGGAGAAGGTGAAATGCT | 192 |
|  |  | R:CCCAGCTCATTTTGTAGTGC |  |
| *NFE2L2* | NM_205117.2 | F: CCCTGCCCTTAGAGATTAGAC | 248 |
|  |  | R: CAAGTTCATGTCCTTTTCTCTGC |  |
| *TRPV1* | XM_046902287.1 | F: GCTTTTGGTCACAGCACTGG | 441 |
|  |  | R: GCTATTTGTGTCCTGCCCCT |  |
| *TRPV2* | XM_040687364.2 | F: AATGCTGGGAGCCATGCTAT | 494 |
|  |  | R: GGAGACATAAAAACAGCAGCCC |  |
| *TRPV3* | XM_040687357.2 | F: CAGCATACACCGAAGAGGCA | 239 |
|  |  | R: TGTTGGTCCTGGTGTTGTCC |  |
| *TRPV4* | NM_204692.2 | F: CCCACCGTACCCCTACACTA | 246 |
|  |  | R: ACCATGACAGCCAGGTAAGC |  |
| *TRPA1* | NM_001318460.2 | F: TGCAATGATGCTGGGAGACA | 191 |
|  |  | R: GAGCGGCGTATTTTTGCACT |  |
| *BNDF* | XM_015286149.2 | F: TTCTCACATGATGACTTCAAACAAG | 185 |
|  |  | R: GCAACTGAAGTATGAGATAACC |  |
| *Eif2b5* | XM_040678751.2 | F: GA AATC CAAGTGGTG CCG | 99 |
|  |  | R: GCATCAACATCTCGCAGCA |  |
| *CREB1* | NM_204450.2 | F: AGTGGAGATGCAGCCGTTAC | 190 |
|  |  | R: GCTGGGCAGCCTGAATTAC |  |
| *CRHR1* | NM_204321.1 | F: TCTGCATTGGCTGGTGTATC | 80 |
|  |  | R: GCACTTCTCGTTGTCGTAGTACAG |  |
| *CRHR2* | XM_015281045.2 | F: AAGAGGAAGTATGCGCACCA | 239 |
|  |  | R: CGACACCAGGGTTCATTGCT |  |
| *DNMT3A* | XM_046914226.1 | F: GGATAGCCAAGTTCAGCAAAG | 145 |
|  |  | R: GGGAAGCCAAACACCCTCT |  |
| *DNMT3B* | XM_046930784.1 | F: GTGCTGTGCCTTGAACATTG | 122 |
|  |  | R: TTCGTAACTTCGGAAACCATT |  |
| *TDG* | XM_046906619.1 | F: GTTTCGAGAAGGAGGGCGAA | 224 |
|  |  | R: CACGAGGGAACTGAGCACAT |  |
| *Gadd45b* | XM_046933968.1 | F: CTGCTGCGACAATGACATCC | 175 |
|  |  | R: GCAGTAATTCGCCACCTCGG |  |
| *EZH2* | XM_046912581.1 | F: TTCAGAGGGAGCAAAGCCTG | 291 |
|  |  | R: TTGGTGTGCTAGGTCTGCTG |  |
| *H3K27* | NM_001031482.3 | F: TTGATCCGCAAGCTTCCCTT | 128 |
|  |  | R: AGGCCAACCAAGTAGGCTTC |  |
| *SLC3A1* | XM_040667709.2 | F: GGCTTACCAATGGAGCTGAGTGAG | 100 |
|  |  | R: CTTGGCTGCTGGTGTCAGTATCC |  |
| *SLC6A14* | XM_040670974.2 | F: CTCCAGTGGGCTGGATGAGA | 88 |
|  |  | R: CAAGGCAGCTCCAACGATCA |  |
| *FBP1* | NM_001278048.2 | F: ATGACCCGCTTCGTGATG | 146 |
|  |  | R: CCAGCAATCCCATACAGGTT |  |
| *ACP6* | XM_046907869.1 | F: GATGCAGCAGATGTTTGCCC | 279 |
|  |  | R: TCCTTCAACTTCTGCCTGGTC |  |
| *FOXO1* | NM_204328.2 | F: TGGGTGTCAGGCTAGGATGT | 196 |
|  |  | R: GCTGCCAACTCTGACGAAAG |  |
| *DIO3* | NM_001122648 | F: CAGCCACGCTCTGTCAATAC | 138 |
|  |  | R: AGATCCCGAAGGAAGAGAGC |  |
| *IGF1* | NM_001004384.3 | F: CAGATAGAGCCTGCGCAATG | 309 |
|  |  | R: GCACCACACAGTGTTTCTGG |  |
| *IGF1R* | NM_205032.3 | F: GTCCAGGAACGATGGAGGAG | 127 |
|  |  | R: CAGGCTCTCTTGCCACATGA |  |
| *IGF2* | NM_001030342.5 | F: ACAGAAGTTGAGAGCGGCAG | 384 |
|  |  | R: CTTCTGGAAGCTCTCCTTGTTG |  |
| *GHR* | XM_046934919.1 | F: ATGGAAGGAGTTAGAACCCA | 164 |
|  |  | R: ATTCAATGCCTGCTTGAGTA |  |
